# Identification and classification of genes regulated by phosphatidylinositol 3-kinase- and TRKB-mediated signalling pathways during neuronal differentiation in two subtypes of the human neuroblastoma cell line SH-SY5Y

**DOI:** 10.1186/1756-0500-1-95

**Published:** 2008-10-28

**Authors:** Yuichiro Nishida, Naoki Adati, Ritsuko Ozawa, Aasami Maeda, Yoshiyuki Sakaki, Tadayuki Takeda

**Affiliations:** 1 Computational and Experimental Systems Biology Group, RIKEN Genomic Sciences Centre, 1-7-22 Suehiro-cho, Tsurumi-ku, Yokohama, Kanagawa 230-0045, Japan

## Abstract

**Background:**

SH-SY5Y cells exhibit a neuronal phenotype when treated with all-trans retinoic acid (RA), but the molecular mechanism of activation in the signalling pathway mediated by phosphatidylinositol 3-kinase (PI3K) is unclear. To investigate this mechanism, we compared the gene expression profiles in SK-N-SH cells and two subtypes of SH-SY5Y cells (SH-SY5Y-A and SH-SY5Y-E), each of which show a different phenotype during RA-mediated differentiation.

**Findings:**

SH-SY5Y-A cells differentiated in the presence of RA, whereas RA-treated SH-SY5Y-E cells required additional treatment with brain-derived neurotrophic factor (BDNF) for full differentiation. After exposing cells to a PI3K inhibitor, LY294002, we identified 386 genes and categorised these genes into two clusters dependent on the PI3K signalling pathway during RA-mediated differentiation in SH-SY5Y-A cells. Transcriptional regulation of the gene cluster, including 158 neural genes, was greatly reduced in SK-N-SH cells and partially impaired in SH-SY5Y-E cells, which is consistent with a defect in the neuronal phenotype of these cells. Additional stimulation with BDNF induced a set of neural genes that were down-regulated in RA-treated SH-SY5Y-E cells but were abundant in differentiated SH-SY5Y-A cells.

**Conclusion:**

We identified gene clusters controlled by PI3K- and TRKB-mediated signalling pathways during the differentiation of two subtypes of SH-SY5Y cells. The TRKB-mediated bypass pathway compensates for impaired neural function generated by defects in several signalling pathways, including PI3K in SH-SY5Y-E cells. Our expression profiling data will be useful for further elucidation of the signal transduction-transcriptional network involving PI3K or TRKB.

## Background

SH-SY5Y cells are the third successive subclone of the SK-N-SH human neuroblastoma cell line [1]. These cells arrest in the G1 phase and exhibit a distinct neuronal phenotype when treated with RA [2]. Morphological changes and expression of biochemical and functional neural markers in SH-SY5Y cells treated with RA resemble those of neurons. SH-SY5Y cells are thus used as a model system for studying the molecular mechanisms involved in neuronal differentiation [3-5].

In SH-SY5Y cells, the PI3K/AKT signalling pathway activated by RA is important for the regulation of neuronal survival and differentiation [6]. In addition, RA promotes the activation of PI3K, leading to the activation of a Rho GTPase, RAC1, that is implicated in the activation of MAPKs, expression of neural markers and neurite outgrowth in SH-SY5Y cells [7]. RA treatment of SH-SY5Y cells also induces expression of TRKB (NTRK2), but not of TRKA (NTRK1), and mediates biological responsiveness to receptors for the neurotrophins BDNF and NT-4/5 [8]. Additional treatment of SH-SY5Y cells with BDNF stimulates tyrosine phosphorylation of TRKB [9], followed by activation of the PI3K/AKT and Ras/MAPK pathways, and the promotion of cell survival and neurite outgrowth in serum-free medium [8,10].

Although the activation mechanisms of signalling pathways stimulated by RA and the neurotrophin have been extensively studied, the link between these pathways and the downstream transcriptional network controlling the expression of target genes required for differentiation of SH-SY5Y cells remains unclear. To examine these mechanisms, we compared the gene expression profiles in SK-N-SH cells and two subtypes of SH-SY5Y cells (SH-SY5Y-A and SH-SY5Y-E), each of which display a different morphology during RA-mediated differentiation.

## Methods

### Cell culture

A protocol including 15% FBS in the culture condition has been previously described [10] as a method for sequential treatment of SH-SY5Y cells with RA and BDNF, although the present study used a D-MEM/F12 1:1 mixture medium, as recommended in the product information sheets. Briefly, random cultured cells from two clone subtypes of SH-SY5Y and SK-N-SH were seeded on laminin-coated culture dishes (BD Bioscience) for 1 day, and were then transferred to medium containing 10 μM RA in the presence or absence of LY294002 (10 μM) for 5 days. For BDNF-induced sequential differentiation of SH-SY5Y-E cells, cells were washed with D-MEM/F12 twice after 5 days and were then incubated with 50 ng/ml BDNF in D-MEM/F12 without serum for 3 days.

### Total RNA preparation

For microarray experiments, total cellular RNA was extracted from cells at specific intervals using a RiboPure Kit (Ambion) in accordance with the manufacturer's instructions. The quality of total RNA was assessed using an Agilent 2100 Bioanalyzer (Agilent Technologies). RNA samples were prepared at least twice for each cell line and each time point, were then stored at -80°C.

### Oligonucleotide microarray (GeneChip) analysis

Microarray analysis was conducted according to the manufacturer's instructions (Affymetrix) and was performed at least twice in order to confirm the reproducibility of gene expression profiles.

### Computational analysis of microarray data

Mean signal intensity for all probes was initially tuned to 500 as global scaling and individual signal intensities were evaluated by a detection call (present/marginal/absent) using Affymetrix MicroArray Suite 5.0 software (Affymetrix). Absent or marginal detection was judged based on a detected transcript being unreliable or suspicious. Signal intensities for all defective probes meeting this criterion were thus tuned to 100, the average signal intensity of these probes. As most probe sequences were designed for the 3' regions of genes [11], each signal intensity was normalised using a normalisation factor of 30,000, the signal intensity of the probe (AFFX-HUMGAPDH/M33197_3_at) derived from the 3' region of GAPDH. All probes on the U133 Plus 2.0 Array were mapped on the human genome (NCBI Build 36.1) with BLAT [12], and probes with sequences that matched RefSeq mapping data [13] were selected. We utilised 29,473 reliable target probes that mapped to their target genes using the Affymetrix formula annotation. Microarray data related to this study are available from the GEO database (accession number: GSE9169) [14].

## Results and discussion

### Phenotypic differences between two subtypes of SH-SY5Y

**Figure 1:**
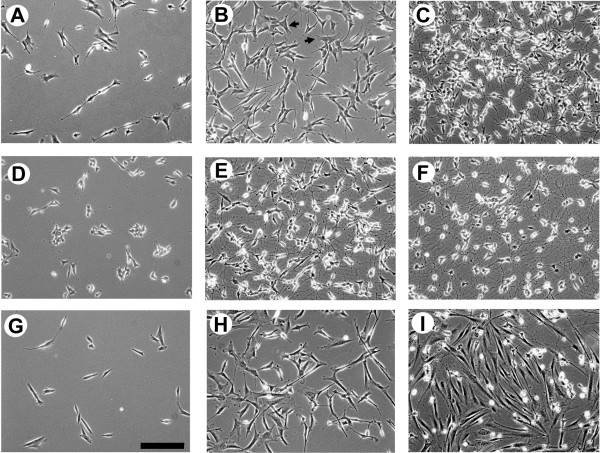
**Morphological changes in two subtypes of SH-SY5Y and SK-N-SH under differentiation conditions.** The panels show SH-SY5Y-E cells (A-C), SH-SY5Y-A cells (D-F) and SK-N-SH cells (G-I). The cells are nondifferentiated (A, D and G), treated for 5 days with 10 μM RA (B, E and H), and sequentially treated with BDNF in the absence of serum for 5 days in SH-SY5Y-E cells (C) or for 3 days in SH-SY5Y-A (F) and SK-N-SH (I) cells. Arrows indicate S-type cells. Phase contrast microscopy ×100. Scale bar represents 200 μm. Note that RA-treated SK-N-SH cells gradually die in the absence of serum (I).

We observed clear differences in the RA-induced neuronal phenotype of the two SH-SY5Y subclones, SH-SY5Y-A and SH-SY5Y-E, which were obtained from two different bioresource centres (ATCC and ECACC, respectively). After 5 days of RA treatment, ECACC (SH-SY5Y-E) cells were slightly larger in size (Fig. 1A) and contained a significant amount of neuroblastic (N-type) cells and a small fraction of epithelial (S-type) cells (Fig. 1B). The appearance of S-type cells was suppressed by RA-mediated differentiation on laminin, which promotes neurite outgrowth of SH-SY5Y cells [15]. Full differentiation of RA-treated cells required sequential treatment with BDNF (Fig. 1C), thus supporting previous findings [10]. Conversely, ATCC (SH-SY5Y-A) cells easily differentiated into a neuronal phenotype with extended neurites and formed a neural network after 5 days of RA treatment (Fig. 1E), again supporting previous observations [16]. The neuronal phenotype of SH-SY5Y-A cells was further enhanced by sequential treatment with RA and BDNF in serum-free medium for 3 days (Fig. 1F). The original SK-N-SH human neuroblastoma cell line comprises two different cell types: a large fraction of epithelial Schwann cells (S-type cells); and a small number of neuroblast cells (N-type cells) [17]. No neural network is formed by the larger fraction of SK-N-SH cells (> 80%) in the presence of RA (Fig. 1H). Instead, these cells elongate and gradually undergo cell death with sequential BDNF treatment under serum-free conditions (Fig. 1I).

### Identification of expressed genes by using microarray analysis

In order to identify the genes required for progression of neuronal differentiation in RA-treated neuroblastoma cells, we first performed a microarray analysis of global gene expression profiles for the two subtype clones of SH-SY5Y and SK-N-SH cells treated with RA for 5 days. We also included a perturbation experiment using a potent PI3K inhibitor, LY294002, to impair RA-induced differentiation of SH-SY5Y cells (data not shown) [6,7]. For full differentiation of RA-treated SH-SY5Y-E cells, gene expression profiles were also analysed following sequential BDNF treatment of cells for an additional 3 days [10].

**Figure 2:**
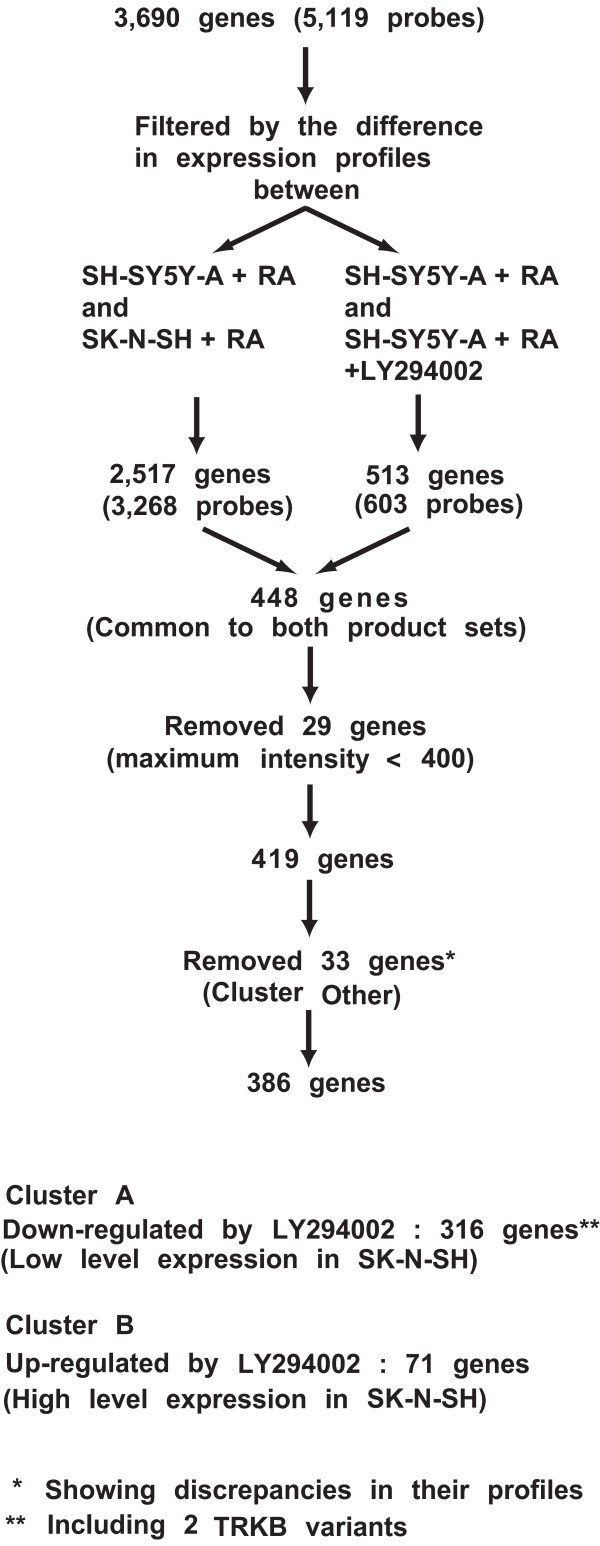
**Classification of genes regulated by the PI3K signalling pathway.** A flow chart summary of how the genes (probes) were categorised into two clusters by statistical analysis of the microarray data. Cluster A includes genes down-regulated by LY294002 in RA-treated SH-SY5Y-A cells and those maintained at a low level in RA-treated SK-N-SH cells. Conversely, cluster B contains genes up-regulated by LY294002 in SH-SY5Y-A cells and maintained at a high level in SK-N-SH cells.

We selected 3690 genes (5119 probes) for comparative analysis of gene expression profiles. These genes were extracted by combining 3100 genes (3628 probes) derived from a sum of the top 1000 probes exhibiting significant expression patterns by ANOVA [18,19] under the 5 culture conditions described above, with 1059 genes (1950 probes) identified as being more significant (Fig. 2).

### Classification of genes regulated by the PI3K signalling pathway

We first compared the differential gene expression profiles of SH-SY5Y-A cells and SK-N-SH cells, because there is a clear phenotypic difference between these cell lines under RA-treated conditions (Fig. 1). Two-factor ANOVA has previously been used for the statistical analysis of normalised data in order to determine differences in gene expression between cell lines and time points [20]. As summarised in Figure 2, we identified a gene cluster containing 2517 genes with significantly different expression profiles (*p *< 0.001) between SH-SY5Y-A cells and SK-N-SH cells treated with RA for 5 days. We also identified 513 genes with expression levels that were significantly down- or up-regulated when PI3K was inhibited in RA-treated SH-SY5Y-A cells. Interestingly, 448 of these genes (87.3%) were common in the product sets of gene clusters selected on the basis of differences in expression pattern between SH-SY5Y-A and SK-N-SH. Moreover, expression behaviours of the selected genes were almost identical to those in RA-treated SK-N-SH cells. By removing genes with contradictory profiles or low expression levels, we finally identified gene clusters A and B, comprising 386 genes, and a third "other" cluster with 33 genes regulated by the PI3K signalling pathway in RA-treated SH-SY5Y-A cells.

**Figure 3:**
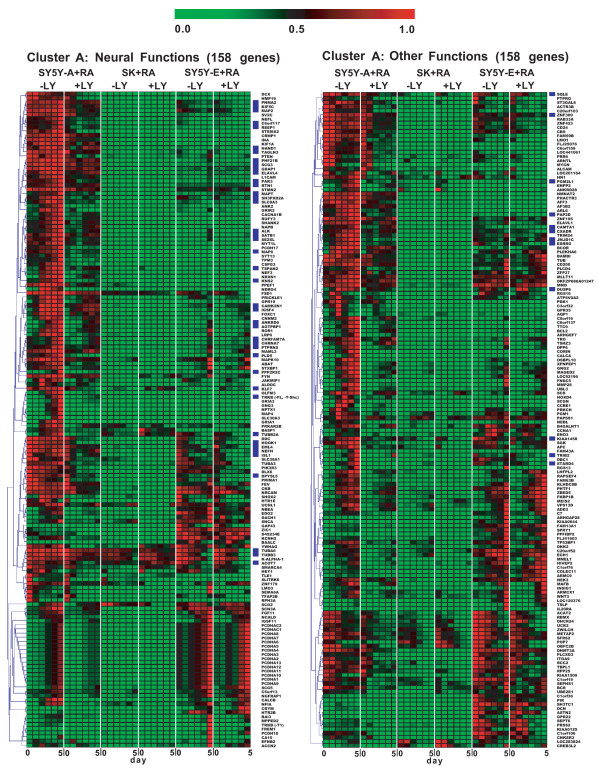
**Gene expression profiles during RA-mediated differentiation.** Gene expression levels were examined at six time points (0 h, 6 h, 1 day, 2 days, 3 days and 5 days) after addition of RA to each neuroblastoma culture. Heat map representation of genes differentially expressed in the indicated cell lines stimulated with RA is shown in the presence or absence of a PI3K inhibitor. Genes are clustered according to hierarchical clustering with Pearson’s correlation. A colour-coded scale (green, down-regulation; red, up-regulation) for percentage expression is indicated at the top of the figure. Cluster A includes genes that were down-regulated by LY294002 in RA-treated SH-SY5Y-A cells. Genes in the cluster were further classified into two subgroups: neural genes; and genes related to other functions. Genes selected in Figure 6 are marked in blue on the right side of the heat map of cluster A.

**Figure 4:**
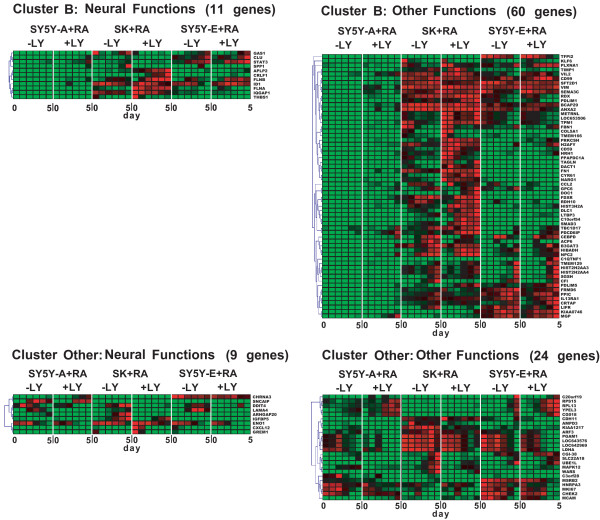
**Gene expression profiles during RA-mediated differentiation (continued). **Cluster B includes genes that were up-regulated by LY294002 in SH-SY5Y-A cells. Cluster “Other” includes 33 genes with contradictory expression profiles between RA-treated SH-SY5Y-A cells with LY294002 and RA-treated SK-N-SH cells. Genes in each cluster were further classified into two subgroups: neural genes; and genes related to other functions.

Figures 3 and 4 show a heat map of expression profiles for all genes in the clusters. We calculated relative signal values (0.000–1.000) of gene expression levels in each cluster under all culture conditions, as summarised in Additional File 1. Cluster A comprises 316 genes with down-regulated expression in RA-treated SH-SY5Y-A cells cultured with LY294002 or extremely low-level expression in RA-treated SK-N-SH cells. Cluster B includes 71 genes with up-regulated expression in RA-treated SH-SY5Y-A cells cultured with PI3K inhibitor or high-level expression in SK-N-SH cells (Figs. 2, 3 and 4 ). Half of the genes in cluster A showed average signal values < 0.1 in RA-treated SK-N-SH cells, too low for quantitative comparison (Additional File 1). To address the effect of a PI3K inhibitor on gene expression in the two clusters, we compared the average signal values of gene expression levels greater than 0.179, the average signal value in the clusters, between RA-treated SK-N-SH cells in the presence and absence of LY294002. In cluster A, the average signal values of 32 of 77 (42%) gene expression levels did not dip below 75% of the average values when RA-treated SK-N-SH cells were exposed to LY294002. Similarly, the average signal values in 35 of 65 (54%) gene expression levels in cluster B did not exceed 125% of the average values by the addition of a PI3K inhibitor (Additional file 1). These findings are consistent with a substantial defect in transcriptional regulation mediated by the PI3K signalling pathway in RA-treated SK-N-SH cells.

We further categorised genes into two subgroups of neural functions and other functions (Figs. 3 and 4), on the basis of Gene Ontology (GO) annotation [21,22] and a comprehensive search of the literature [22-24]. Genes with neural functions were defined when significant GO term(s) and/or literal description(s) implicating involvement in neural events were obtained by the gene annotation. We also defined genes with other functions when neither the GO term nor the literal description was related to neural events. GO annotation in two clusters yielded the identification of 114 genes with GO terms related to neural events (Additional File 2). Cluster A genes included 158 genes in the neural function subgroup and 158 genes in the other functions subgroup. Cluster B genes were similarly classified, with 11 genes in the neural function subgroup and 60 genes in the other functions subgroup (Fig. 3 and 4).  Neural functions were further divided into 9 functions, as summarised in Additional File 2.

### Differences in PI3K-mediated transcriptional regulation between two subtypes of SH-SY5Y

**Figure 5:**
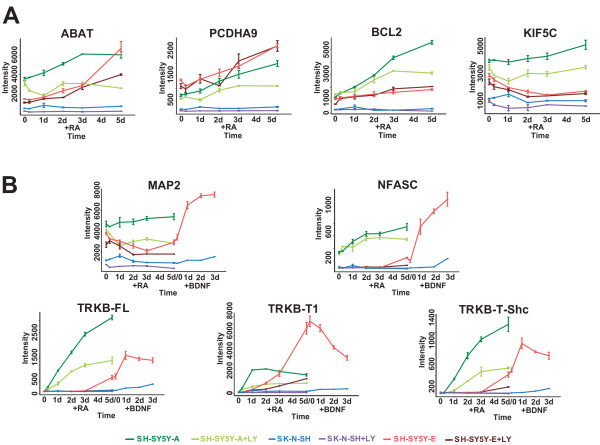
**Typical gene expression profiles of genes identified in this study.**A) Typical gene expression profiles in RA-treated SH-SY5Y-E cells. ABAT, which is partially regulated by the PI3K signalling pathway, and PCDHA9, BCL2 and KIF5C, which are independently regulated by the PI3K signalling pathway, are included in cluster A. B) Typical expression profiles of genes such as MAP2 (left upper panel), NFASC (right upper panel) and TRKB variants (bottom panels), which were categorised as dependent on the PI3K signalling pathway in RA-treated SH-SY5Y-A cells.

To investigate whether the PI3K signalling pathway is sufficiently activated in RA-treated SH-SY5Y-E cells, we further focused on the transcriptional regulation of the 386 genes included in these clusters. Differences in expression profiles of RA-treated SH-SY5Y-E cells with and without LY294002 were compared to differences in expression profiles of RA-treated SH-SY5Y-A cells with and without LY294002 (Fig. 3 and 4). Neurite outgrowth induced in RA-treated SH-SY5Y-E cells was also inhibited by LY294002, thus suggesting that the PI3K signalling pathway is functional in SH-SY5Y-E cells. However, most of the gene expression in the clusters was regulated in a PI3K-independent manner, and half of the neural genes were expressed at extremely low levels (Figs. 3, 4 and 5A), whereas transcriptional inhibition by LY294002 exclusively emerges after 2–3 days in the presence of RA (Fig. 5A, 5B; bottom panels). These results suggest that the PI3K signalling pathway is partially disrupted in RA-treated SH-SY5Y-E cells, with this disruption leading to reduced differentiation. These results also suggest that supplementation with an additional TRKB signalling pathway is required for full differentiation of SH-SY5Y-E cells after down-regulation of the gene cluster required for neural events.

Human TRKB is alternatively spliced into at least 3 variants: TRKB-FL; TRKB-T1; and TRKB-T-Shc [25]. TRKB-FL is a tyrosine kinase-containing variant, whereas the intracellular tyrosine kinase domain is truncated in the other proteins [26,27]. Two typical gene expression profiles were observed by microarray analysis in accordance with different transcriptional regulation on the alternative promoters (Fig. 5B; bottom panels) [28]. In SH-SY5Y-A cells, TRKB-FL and TRKB-T-Shc variants showed continuously induced transcription over 5 days, whereas gene expression of TRKB-T1 was initially induced but plateaued 1 day after RA treatment. In SH-SY5Y-E cells, expression of all TRKB variants, particularly TRKB-T1, was abruptly induced 3 days after RA treatment and peaked after 5 days in the presence of RA (Fig. 5B; bottom panels), thus supporting previous results [29]. These TRKB variants were also transcriptionally regulated by the PI3K signalling pathway (Fig. 5B; bottom panels), indicating clear cross-talk between the TRKB and PI3K signalling pathways. Conversely, induction of TRKB variants was not observed in SK-N-SH cells at any time, providing a possible explanation for the defect in the neuronal phenotype of these cells (Fig. 5B, bottom panels).

### Transcriptional compensation by an additional TRKB-mediated signalling pathway in SH-SY5Y-E

**Figure 6:**
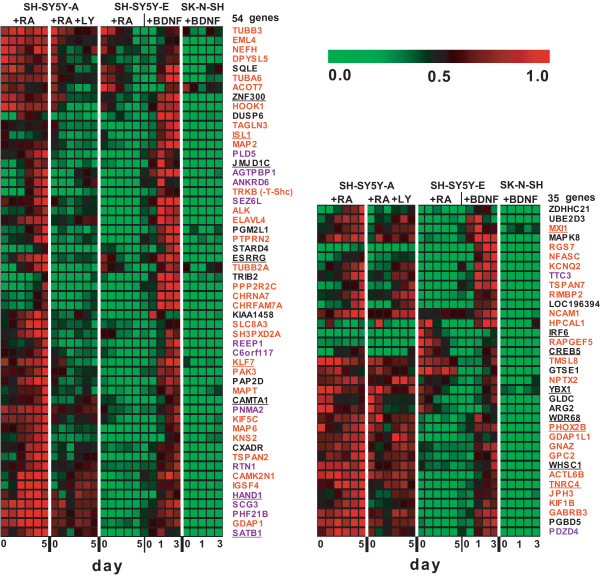
**Expression profiles of genes induced by sequential treatment with BDNF in SH-SY5Y-E cells.** Heat map representation is shown for genes that are down-regulated or maintained at a low level in the presence of RA but are induced by additional treatment with BDNF in SH-SY5Y-E cells. RNA sampling was performed at five time points (0 h, 6 h, 1 day, 2 days and 3 days) after cells were transferred to serum-free medium containing BDNF. To identify the gene expression induced by BDNF, we focused on genes with expression levels that were higher in SH-SY5Y-A cells during differentiation, rather than those in SH-SY5Y-E or SK-N-SH cells. We also selected genes with expression levels in SH-SY5Y-A cells treated with RA for 5 days that exceeded 66.7% of that on day 0, while excluding genes with expression levels that exceeded the 66.7% threshold in SH-SY5Y-E cells treated with RA for 5 days. We selected genes with expression levels that underwent a >1.5-fold change and exceeded the 66.7% threshold when SH-SY5Y-E cells were treated sequentially with BDNF for 3 days. Finally, genes exhibiting similar up-regulation by BDNF in SH-SY5Y-E cells and SK-N-SH cells were also excluded. Genes regulated by the PI3K signalling pathway in RA-treated SH-SY5Y-A cells (left panel) and genes not regulated by the PI3K signalling pathway in SH-SY5Y-A cells (right panel) are shown. The relative alteration of gene expression in these gene pools is summarised in Additional file 1. Genes involved in neural processes are shown in red, and those expressed strongly in brain are shown in purple. Genes encoding transcription factors are underlined.

Additional BDNF treatment of SH-SY5Y cells stimulates tyrosine phosphorylation of TRKB [9], promoting neurite outgrowth in serum-free medium [8,10]. Supporting these findings, additional exposure of RA-treated SH-SY5Y-E cells to BDNF resulted in full differentiation and induced 89 genes that were down-regulated or maintained at low expression levels with RA treatment only (Figs. 5B, 6). These genes were further sorted into two gene pools: 54 genes from cluster A (shown in blue in Fig. 3; Fig. 6, left panel) and 35 genes regulated in a PI3K-independent manner (Fig. 6, right panel). As expected, both of these pools contain many genes required for neural events (Fig. 6; Additional File 2), indicating that these genes are critical for TRKB-mediated differentiation of SH-SY5Y-E cells. These results also demonstrate that genes down-regulated either by an impaired PI3K signalling pathway or by another abrogated signalling pathway are transcriptionally compensated for via an additional TRKB-mediated signalling pathway, leading to full differentiation (Additional File 3). However, a significant number of genes down-regulated or maintained at low expression levels in RA-treated SH-SY5Y-E cells did not exceed the threshold (Fig. 6 legend) following additional BDNF treatment (Fig. 3; Additional File 1), thus suggesting that the TRKB-dependent signalling pathway only partially compensates for an impaired PI3K signalling pathway. The TRKB variants TRKB-FL and TRKB-T-Shc were induced and plateaued following sequential treatment of SH-SY5Y-E cells with RA and BDNF. Conversely, TRKB-T1 expression, which was strongly induced by RA treatment of SH-SY5Y-E cells, was abruptly down-regulated after a shift to serum-free medium with BDNF (Fig. 5B, bottom panels), which supports the notion that the dominant-negative function of tyrosine kinase-deficient receptors [30] is totally diminished by induction of functional TRKB-FL. Further studies may be required to identify the components and transcription factors involved in the TRKB and PI3K signalling pathways.

In conclusion, we identified gene clusters that are transcriptionally controlled by two different signalling pathways mediated by PI3K and TRKB during the differentiation of two subtypes of SH-SY5Y cells. These expression profiling data may prove useful in further elucidating the molecular mechanisms regulating the promoter activities of genes required for neuronal differentiation. These promoter activities are mediated by an upstream signal transduction-transcriptional network including PI3K and/or TRKB.

## Abbreviations

RA: all-trans retinoic acid; PI3K: phosphatidylinositol 3-kinase; BDNF: brain-derived neurotrophic factor; TRKA (NTRK1): Tropomyosin-related kinase A; TRKB (NTRK2): Tropomyosin-related kinase B; MAPK: mitogen-activated protein kinase; NT-4/5: neurotrophin-4/5; ATCC: American Type Culture Collection; ECACC: European Collection of Cell Cultures; D-MEM/F12: Dulbecco's modified Eagle's medium/nutrient mixture F12; FBS: Fetal Bovine Serum; ANOVA: analysis of variance.

## Competing interests

The authors declare that they have no competing interests.

## Authors' contributions

TT, YN and NA designed the research plan and AM performed morphological analysis of the neuroblastoma cells. NA and RO conducted the microarray analysis. TT evaluated the results and YN performed statistical analysis of the microarray data. YS and TT supported the work. TT, YN and NA wrote the manuscript.

## Supplementary Material

Additional file 1 Relative change (0.000–1.000) in gene expression profiles under all culture conditions. A relative minimal value (0.000) and a relative maximal value (1.000) were calculated for each gene by using minimal and maximal signal values in a series of gene expression profiles under all culture conditions. The signal value on day 0, average signal value and maximal signal value (or minimal signal value) was calculated for each gene expression profile in all clusters and are shown together with the expression patterns such as up-regulation (up), down-regulation (down) or constant expression (const) on the basis of the average signal value, as compared with ± 25% fluctuation values of the signal value on day 0. Click here for file

Additional file 2 Gene functions for neural genes in each cluster. Genes in the clusters were first analysed and categorised using Gene Ontology (GO) annotation (downloaded in April 2007) [21]. The gene-GO association was obtained from the NCBI Entrez Gene [22]. A comprehensive search of the literature was further performed using the "Summary" and "GeneRIFs" sections in NCBI, Entrez Gene [22], NCBI, Pub Med [23] and the "Research Articles" section in Gene Cards [24]. The 9 categories for gene function are: microtubule-based process, cell adhesion, axon guidance, synaptic events, ion channel, neurite outgrowth, receptor and receptor binding, signal transduction and others. Click here for file

Additional file 3 A possible molecular mechanism required for transcriptional regulation mediated by two different signalling pathways. Genes up-regulated during RA-mediated differentiation in SH-SY5Y-A cells were classified into groups I, II and III. Group I and group II are controlled by the PI3K signalling pathway, whereas group III is regulated by PI3K-independent pathway(s). In RA-treated SH-SY5Y-E cells, most of the genes involved in RA-mediated differentiation are regulated in a PI3K-independent manner. When down-regulated by the impaired PI3K signalling pathway and by a defect in another signalling pathway, these genes are transcriptionally compensated for by an additional TRKB-mediated signalling pathway, leading to full differentiation. Click here for file
