# Supplementary figures and images for: Identification and classification of genes regulated by phosphatidylinositol 3-kinase- and TRKB-mediated signalling pathways during neuronal differentiation in two subtypes of the human neuroblastoma cell line SH-SY5Y
Source: BMC Res Notes. 2008 Oct 28;1:95. doi: 10.1186/1756-0500-1-95 (PMC2615028; doi:10.1186/1756-0500-1-95)

### SH-SY5Y-A cells

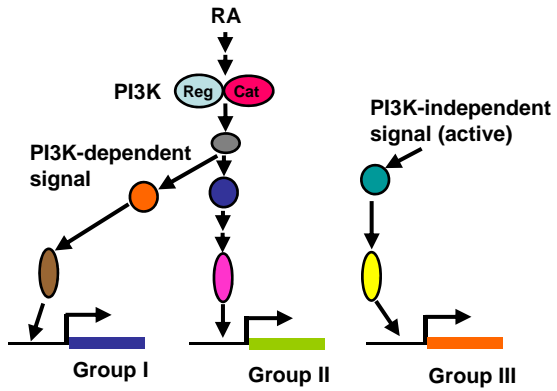

### SH-SY5Y-E cells

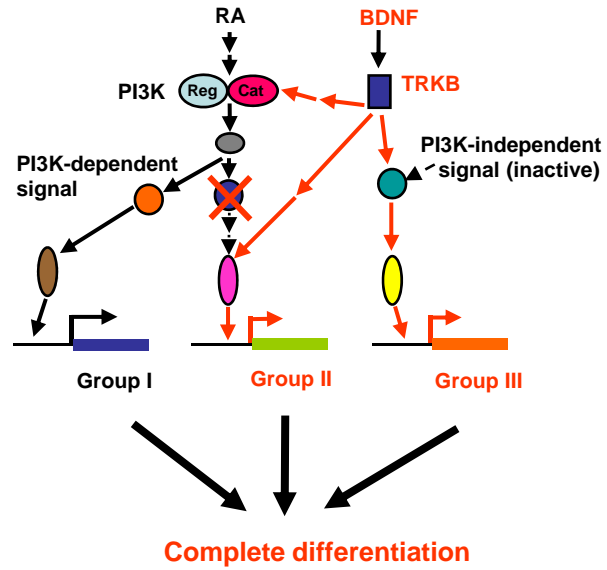

Supplement: Additional file 3 — A possible molecular mechanism required for transcriptional regulation mediated by two different signalling pathways. Genes up-regulated during RA-mediated differentiation in SH-SY5Y-A cells were classified into groups I, II and III. Group I and group II are controlled by the PI3K signalling pathway, whereas group III is regulated by PI3K-independent pathway(s). In RA-treated SH-SY5Y-E cells, most of the genes involved in RA-mediated differentiation are regulated in a PI3K-independent manner. When down-regulated by the impaired PI3K signalling pathway and by a defect in another signalling pathway, these genes are transcriptionally compensated for by an additional TRKB-mediated signalling pathway, leading to full differentiation. [file 1756-0500-1-95-S3.pdf]
